# Antigen presentation by tumor infiltrating B cells influences CD4 T cell phenotype and function in primary lung cancer patient tumors

**DOI:** 10.1186/2051-1426-3-S2-P397

**Published:** 2015-11-04

**Authors:** Tullia Bruno, Peggy Ebner, Brandon Moore, Daniel Munson, John Mitchell, Jeffrey Kern, Dario AA Vignali, Jill Slansky

**Affiliations:** 1 University of Pittsburgh, Denver, CO, USA; 2 University of Colorado School of Medicine, Denver, CO, USA; 3 National Jewish Health, Denver, CO, USA; 4 University of Pittsburgh, Pittsburgh, PA, USA

## 

Despite improvements in surgical techniques and combined chemotherapies, the 5-year survival rate for all stages of non-small cell lung cancer (NSCLC) is only 18%. Understanding the function of tumor infiltrating lymphocytes (TILs) in NSCLC patient tumors will contribute to the development of rationally designed treatments and improved statistics. B cells in tumors (TIL-Bs) are detected in non-small cell lung cancer (NSCLC) and their frequency correlates with improved survival, however, the functional mechanism of TIL-Bs in solid tumors is not well understood. We hypothesize that TIL-Bs help generate potent, long-term immune responses against cancer by presenting tumor antigens to CD4 tumor infiltrating lymphocytes (TILs) in primary human lung tumors.

**Figure 1:**
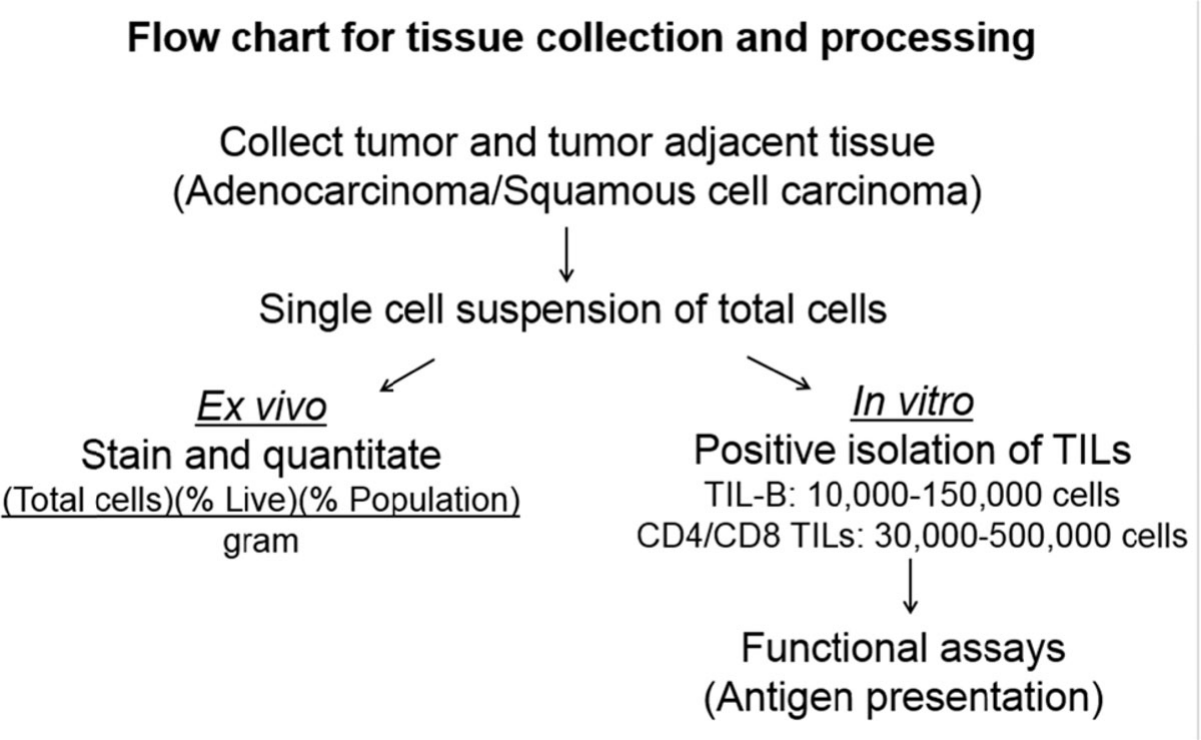


**Figure 2:**
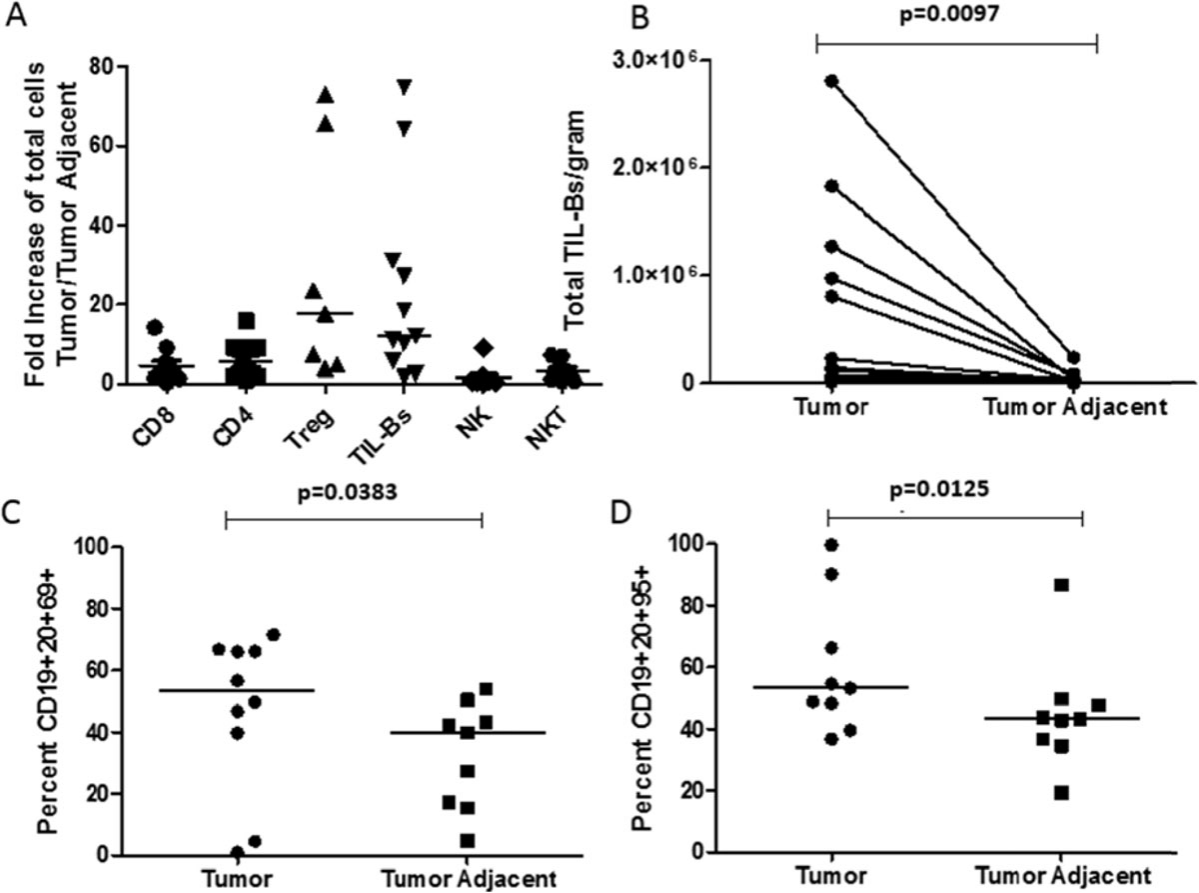


**Figure 3:**
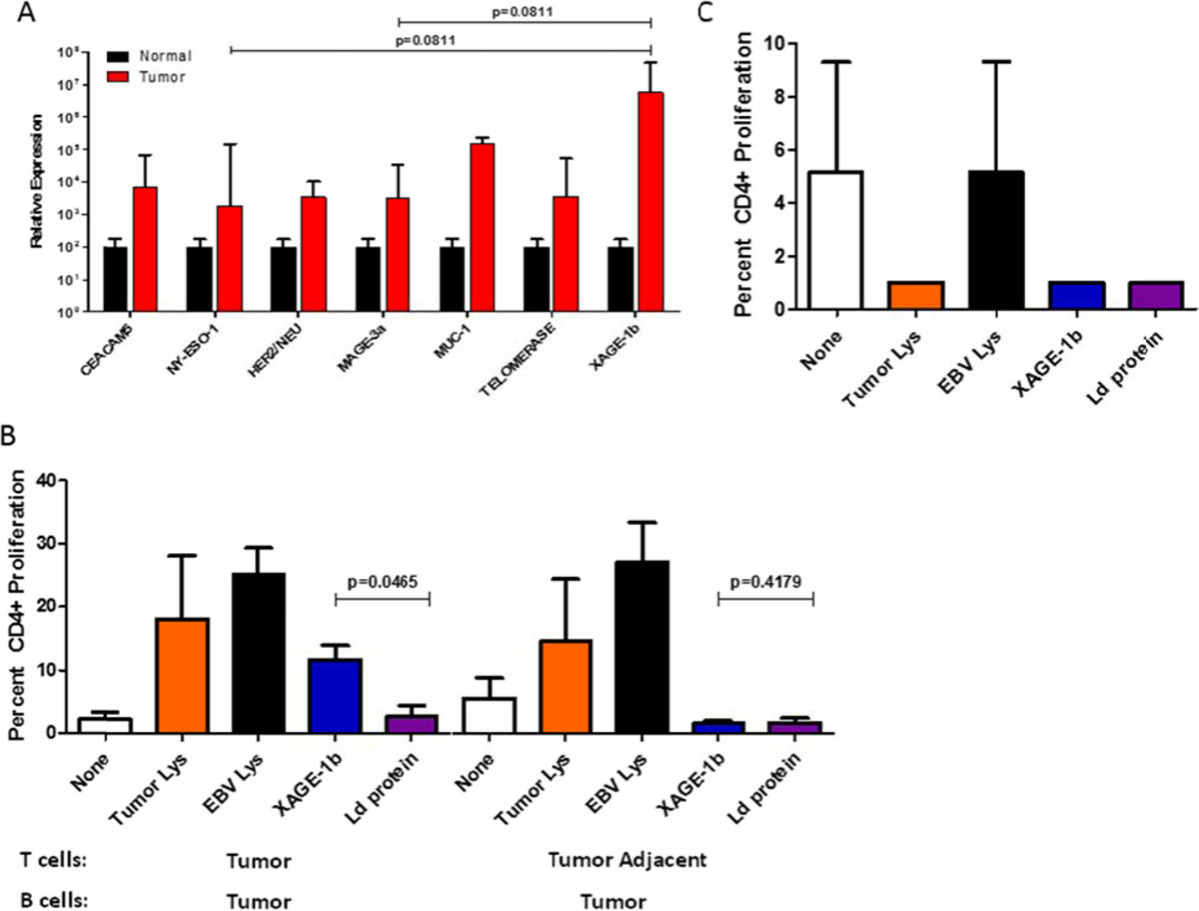


**Figure 4:**
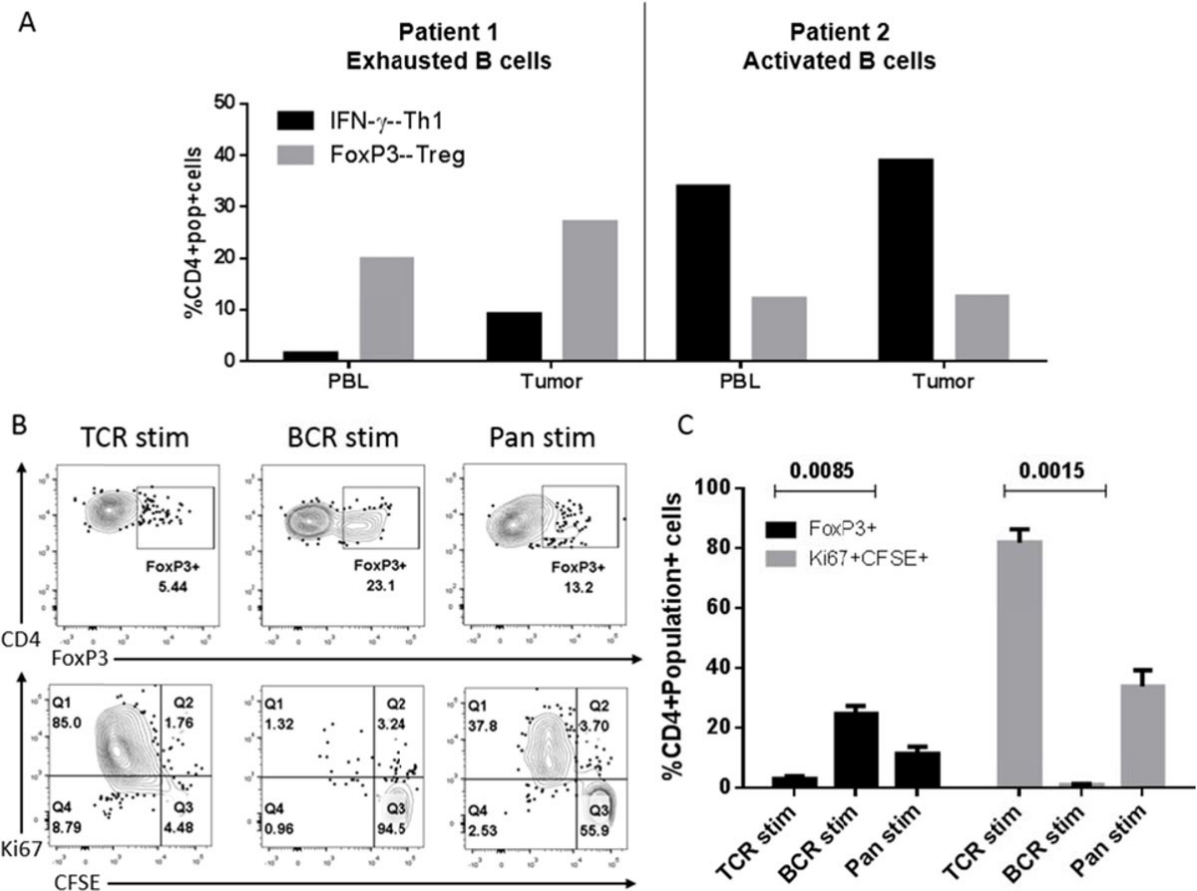


Using un-manipulated, primary human B cells from fresh tumor, tumor-adjacent, and normal (cancer-free) lung tissue per the protocol described in Figure 1, we observed that the total number of B cells at the site of the tumor versus the tumor-adjacent tissue was increased compared to other immune subsets (Figure 2). Further, in analyzing B cell markers of activation and exhaustion, we observed a spectrum of activation of TIL-Bs (Figure 2). Finally, we demonstrated that TIL-Bs present autologous tumor antigens (particularly the cancer-testis antigen XAGE-1b) to CD4 TILs in a subset of NSCLC patients; if the TIL-Bs were activated (CD69+CD27+CD21+) they polarized the CD4 TILs to T helper (anti-tumor) CD4 T cells and if the TIL-Bs were exhausted (CD69-CD27-CD21-CD95+) they polarized the CD4 TILs to T regulatory cells (pro-tumor) (Figure 3 and Figure 4). These data suggest that TIL-Bs polarize the phenotype and function of CD4 TILs in NSCLC patient tumors.

In conclusion, TIL-Bs can have dual function in NSCLC patient tumors, and determining if the TIL-Bs are activated or exhausted will allow appropriate stimulation of the anti-tumor function of TIL-Bs in NSCLC patients. Ultimately, results from this study will help predict how to target TIL-B functions in future TIL-B-specific immunotherapies or in combination with current immunotherapies for NSCLC patients like blockade of the inhibitory receptor, PD-1.

